# Using personas and journey maps as knowledge translation tools to enhance clinicians’ interpretation of PROM scores

**DOI:** 10.1186/s41687-025-00940-y

**Published:** 2025-10-07

**Authors:** Jae-Yung Kwon, Melissa Moynihan, Kayoung Lee, Shauna Remin, Angela C. Wolff

**Affiliations:** 1 School of Nursing, University of Victoria, Victoria, Canada; 2 Institute on Aging and Lifelong Health, Victoria, Canada; 3 School of Nursing, Trinity Western University, Langley, Canada; 4 School of Nursing, University of British Columbia, Vancouver, Canada; 5 BC Cancer, Surrey, Canada

**Keywords:** Person-centred care, Personas, Journey maps, Patient-reported outcome measures, Knowledge translation

## Abstract

Patient-reported outcome measures (PROMs) are common tools for assessing patients’ health, disease condition, functional status, well-being, and quality of life that can achieve person-centred care. While PROMs provide valuable numeric scores, they do not capture contextual depth, thereby making it difficult for clinicians to interpret scores in ways that reflect the complexities of individual lived experiences. This commentary introduces personas and journey maps as educational knowledge translation tools to support a more holistic interpretation of PROM data. Personas integrate PROM data with patient narratives to create relatable archetypes that reflect the values, challenges, and priorities of various patient groups. Journey maps, in turn, visually trace patients’ interactions with the healthcare system over time, identifying key events and transitions that influence their experiences. Together, these tools offer clinicians a story-informed framework to interpret PROM data in ways that are grounded in patient experience. Integrating PROM data within personal and temporal contexts can enhance the relevance, empathy, and practical utility of PROMs for person-centred care.

## Introduction

Healthcare based on a person-centred approach prioritize patients’ experiences, values, and goals, which is a shift from the traditional medical model. One way to incorporate patient needs and experiences is the use of patient-reported outcome measures (PROMs), which are standardized instruments that capture patients’ perceptions of their health, disease condition and its treatment, functional status, mental wellbeing, and overall quality of life. Complementary to clinician-reported outcomes and diagnostic tests, these measures are instrumental in assessing patients, discerning treatment and care options, facilitating shared decision-making, and evaluating healthcare interventions [1]. However, a common barrier to their use in practice is the challenge clinicians face in interpreting PROM scores in meaningful ways [2]. Without adequate context, there is a risk that clinicians may overlook the intricate social and contextual realities that inform PROM responses. PROMs reveal the “what,” but not always the “why” behind patients’ responses. For example, two patients might report similar pain scores on a PROM, yet the meaning and consequences of that pain may differ depending on their cultural background, emotional resilience, or social support [3]. Without sufficient contextual understanding within the therapeutic encounter, clinicians may misinterpret PROM scores, leading to the potential to inhibit patient-clinician interaction and misrepresenting patient issues [4]. To interpret PROM scores, it is important for clinicians to discuss the results with patients to gain insights into each person’s unique context. This commentary introduces two knowledge translation (KT) tools, personas and journey maps, to support the implementation of PROMs by clinicians at the point of care. These innovative tools are co-designed with researchers and patients to situate PROM data within patient narratives for enhancing their interpretation to inform patient care. When using implementation strategies such as education and training [5], personas and journey maps can help improve clinicians’ ability to interpret PROM scores considering patients’ lived realities. In this commentary, we will describe PROM-based personas and journey maps as education/training KT tools, present scenarios, and then examine practical challenges and considerations for integrating these tools to inform person-centred care.

### PROM-based personas for knowledge translation

**Fig. 1:**
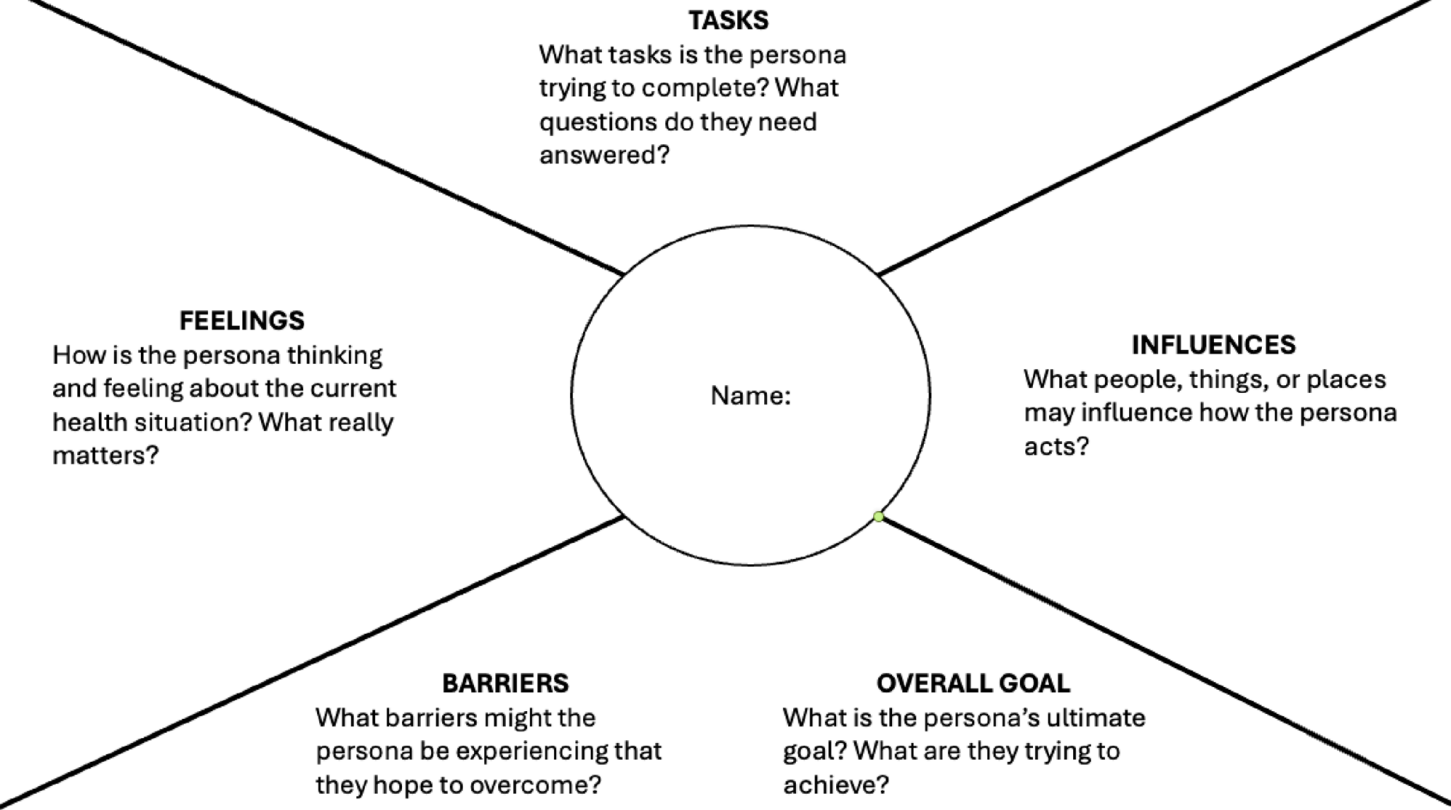
Empathy mapping

Personas are hypothetical yet realistic representations of patients’ lived experiences to capture the emotional, psychological, and social dimensions of care. As KT tools, personas can help bridge the gap between PROMs and the nuanced realities of patients’ lives, translating numeric scores into more meaningful and actionable insights for clinicians. Originally developed in user-centred design and marketing, personas have been adapted in healthcare to enhance understanding of diverse patient populations. Typically, personas are co-designed by researchers and patient partners (i.e., individuals with lived health experience) using qualitative methods such as interviews, focus groups, and participatory workshops. Personas incorporate key demographic and clinical characteristics (e.g., name, age, severity of diagnosis, and treatment) and use empathy mapping techniques to capture what the persona is thinking, feeling, saying, and doing at different stages of their care journey [6]. What distinguishes our approach is the intentional integration of PROM data following the persona development process. This allows for contextualizing PROM scores within a rich narrative that reflects lived experience, thus enhancing interpretability and practical application for clinicians (see Fig. 1).

In our project with older adults living with cancer, we first co-designed personas through empathy mapping and then invited participants to complete a PROM from the perspective of the persona [7]. While various PROMs could be used, we focused on the emotional well-being domain of the FACT-G (Functional Assessment of Cancer Therapy – General), based on both patient priorities and literature highlighting the unmet emotional support needs of older adults during cancer care [8]. Participants were asked to reflect on each item of the PROM in relation to the persona’s attributes (e.g., feelings, tasks, social influences) and to explain why they selected a particular response. This process revealed important clinical insights and unmet patient needs that were not apparent through numeric scores alone, and thereby offered a more holistic and contextual interpretation of PROM data shaped by individual responses.

### PROM-based persona for knowledge translation

**Fig. 2:**
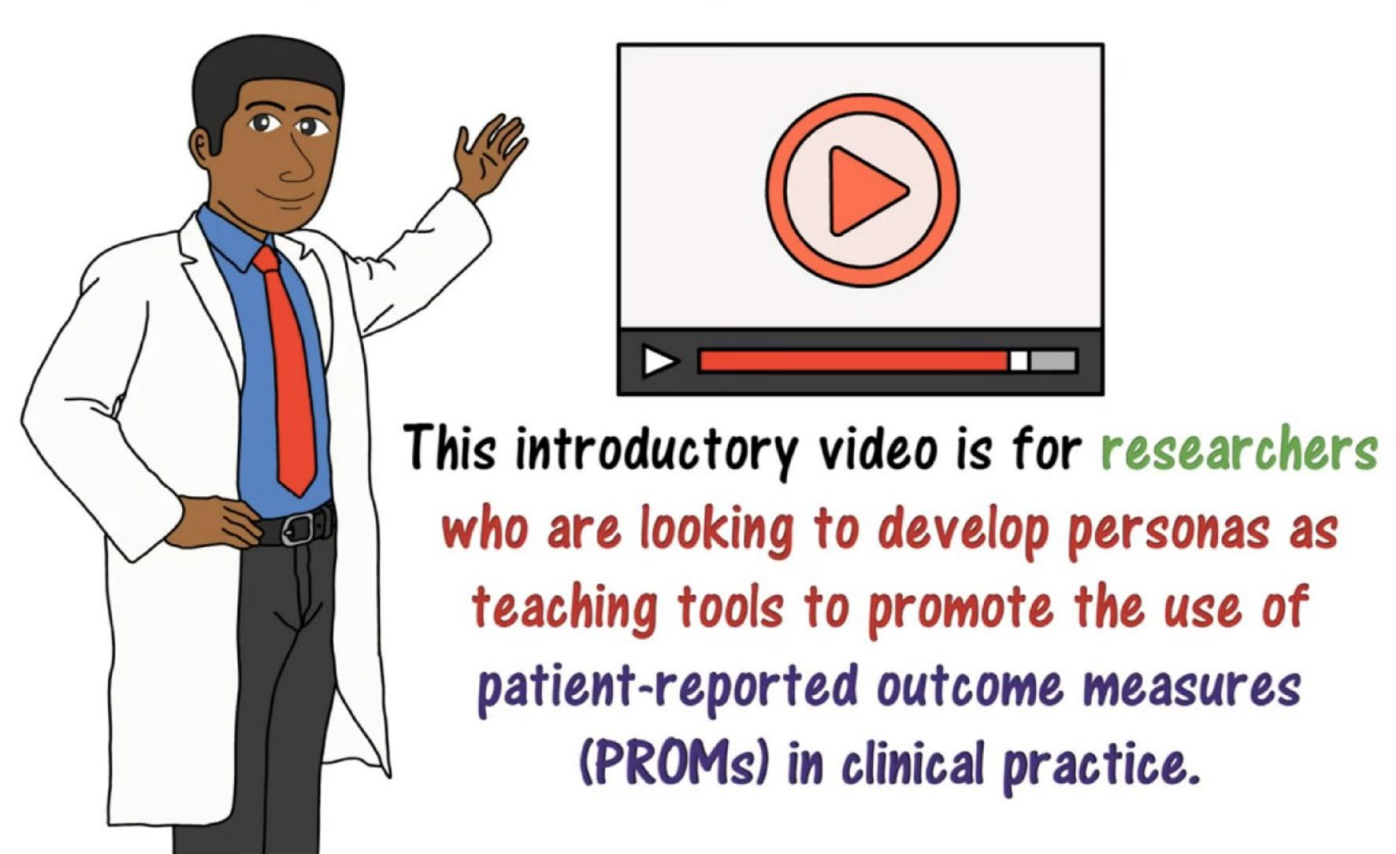
Whiteborad video to illustrate personas as teaching tools. *Adapted from: Kwon*,* J.-Y.*,* Moynihan*,* M.*,* Hawkins*,* B.*,* Yee*,* A.*,* & Irlbacher*,* G. (2022). Personas as teaching tools to promote the use of patient-reported outcome measures in practice [Video]. YouTube.* https://www.youtube.com/watch?v=-mUh6NDEeZQ

Personas serve as meaning-making tools for translating numerical PROM data into relatable patient narratives, helping clinicians understand how patient factors (e.g., life experiences, illness trajectory, social context, and care goals) shape individual responses. By making these underlying influences more visible, personas enable clinicians to interpret PROM scores not as isolated data points but as entry points into patients’ lived realities. This nuanced understanding guides clinicians to ask thoughtful, empathetic questions that foster meaningful dialogue and support individualized care planning. In this way, personas with integrated PROM data act as interpretive bridges that connect standardized measures to individual patient needs, priorities, and experiences. For an overview of how personas are co-designed, see Fig. 2.

#### Persona scenario: “Trish”

**Fig. 3:**
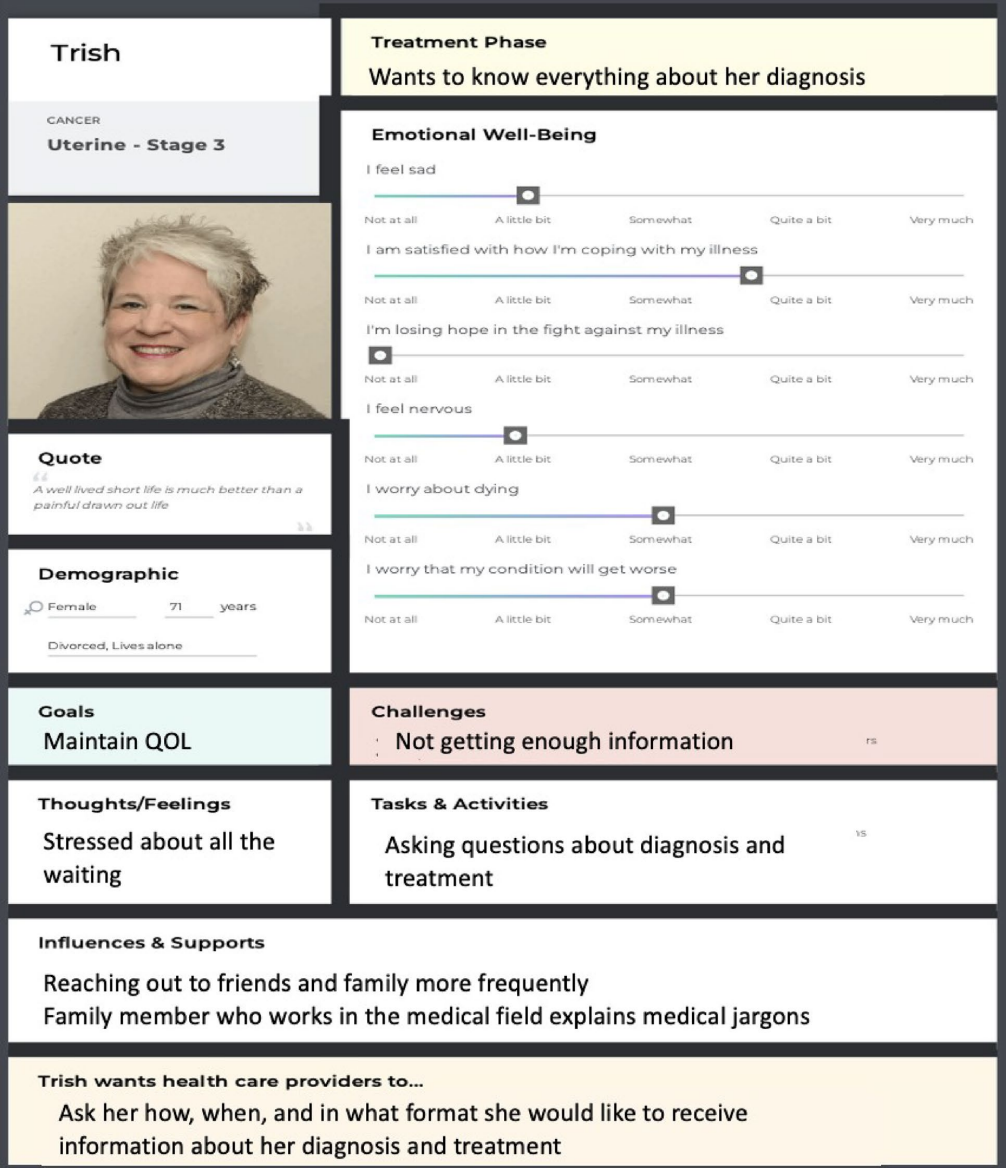
Persona of Trish. Adapted from “Seeing the person before the numbers: Personas for understanding patients’ life stories when using patient-reported outcome measures in practice settings”, by Kwon et al., 2023, *International Journal of Medical Informatics*, 172, 105016. Copyright 2023 by Elsevier

Consider “Trish”, a 71-year-old woman diagnosed with uterine cancer (see Fig. 3). As part of her clinic visit, she completes the FACT-G questionnaire (i.e., a PROM), which includes a section on emotional well-being. In reviewing Trish’s FACT-G responses, the clinician notes that she answered “somewhat” to the item: “I worry that my condition will get worse”. Rather than accepting Trish’s response at face value, the clinician draws on her expertise to probe further, engaging Trish by asking, “I see you are worried about your condition worsening. Can you tell me more about this worry?” Trish shares that she places a higher value on her quality of life than on prolonged survival and that she found it challenging to access clear information about her diagnosis. This insight prompts the clinician to reframe the consultation, instead of focusing solely on clinical indicators or aggressive treatment options, the conversation shifts toward quality of life considerations, including discussions about managing side effects, accessing palliative care, and identifying treatment pathways that align with Trish’s values.

**Fig. 4:**
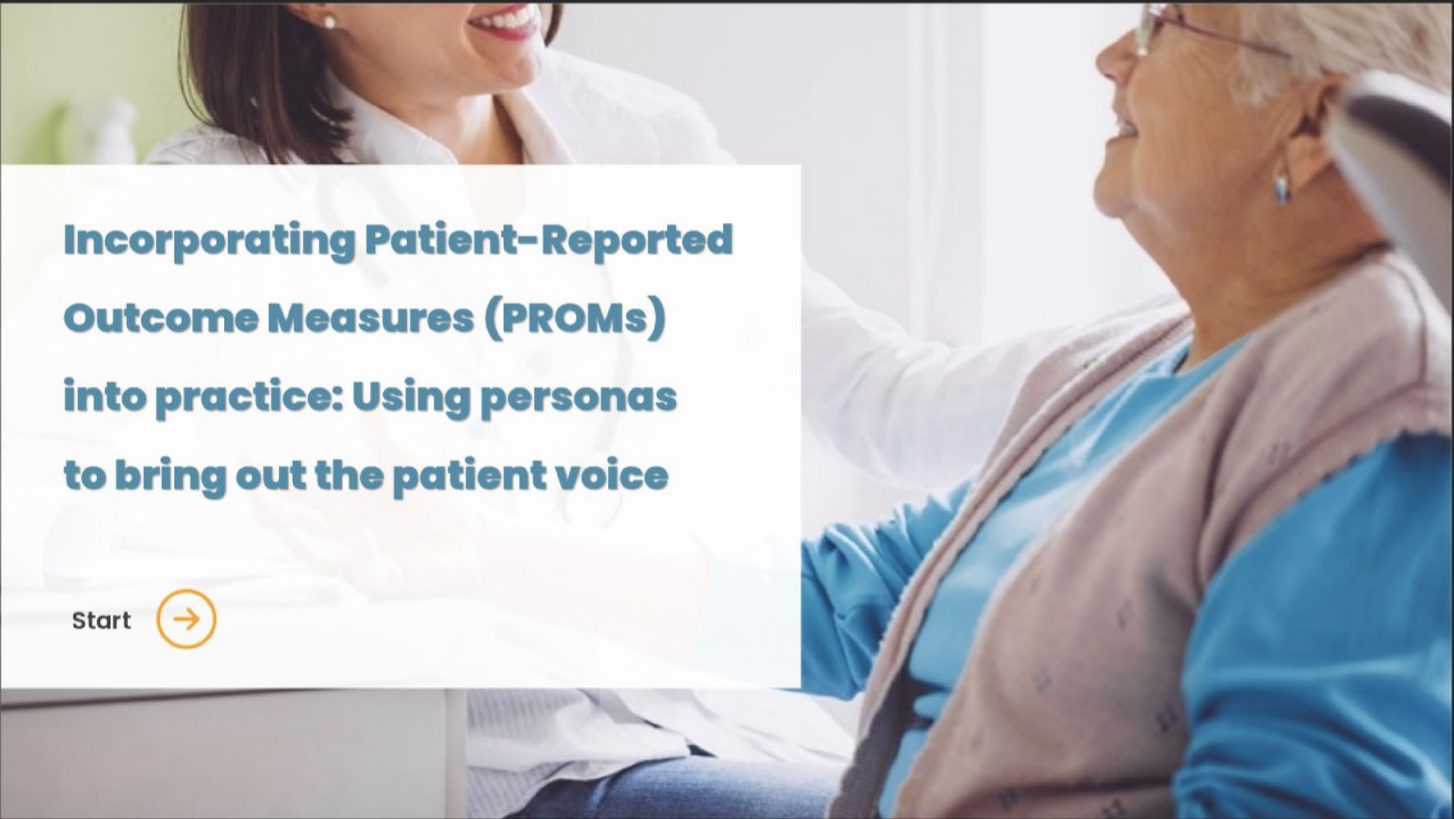
Illustration of an online persona scenario module. Adapted from: Kwon, J.-Y., Lau, F., Sawatzky, R., Wolff, A., Hung, L., Lambert, L., Moynihan, M., Hawkins, B., Yee, A., & Irlbacher, G. (2022, March 14). *Incorporating patient-reported outcome measures into practice: Using personas to bring out the patient voice* [Learning module]. https://www.jykngroup.com/learning-module/story

Scenarios such as this, in which clinicians use PROMs as a springboard for further conversation, illustrates how personas, when strategically used as KT tools to contextualize PROM data, can highlight the need for ongoing patient-clinician communication about PROM results for the clinician to gain a deeper, holistic understanding of patients’ lived experiences and needs. To further support clinicians in using PROMs to facilitate patient-clinician communication, a dedicated practice scenario module is available online (see Fig. 4).

This resource (Fig. 4) offers case-based examples and sample dialogue, demonstrating how PROM data can be meaningfully integrated into clinical encounters using person-centred communication strategies. Used as tools for education and training, personas provide clinicians with the practical experience to learn how PROM scores are used to create a holistic understanding of patients, complementary to clinician-based data and tests, to inform shared decision-making that incorporates patients’ lived experiences, values, and care goals.

### PROM-based journey maps for knowledge translation

Journey maps are a powerful method for visualizing patients’ care needs and goals over time, illustrating key events in patients’ health trajectory [9]. As KT tools, journey maps can support a better understanding of PROMs to improve patients’ care [10]. While journey mapping methods vary, they usually involve gathering qualitative data through in-depth patient interviews. Integrating PROM data with journey maps provide a more contextualized understanding of patients’ health trajectories over time. This approach offers a comprehensive overview of patients’ physical, emotional, and social well-being over time. Journey maps with PROM data can serve as resources for education and training to build capabilities of clinicians to use PROM data for identifying emerging needs and proactively addressing evolving patient concerns. In our project, we developed journey maps that visualized changes in well-being scores over time (i.e., PROM data) from semi-structured interviews with older adults who had undergone radiation therapy for cancer [10]. Individual interviews were conducted via Zoom or telephone to explore participants’ experiences across four phases of care: pre-diagnosis, diagnosis, treatment, and post-treatment. At each phase, participants completed a well-being item from the revised Edmonton Symptom Assessment System (ESAS-r), rating their overall well-being on a scale from 0 (best) to 10 (worst). Participants were then asked why they chose a particular response and used the empathy mapping categories (e.g., feeling/thinking, tasks/activities, and influences) to contextualize their responses. The well-being scores and the empathy mapping categories were subsequently integrated into their personalized journey maps, capturing fluctuations in well-being and associated contextual factors.

#### Journey mapping scenario: “Fiona”

**Fig. 5:**
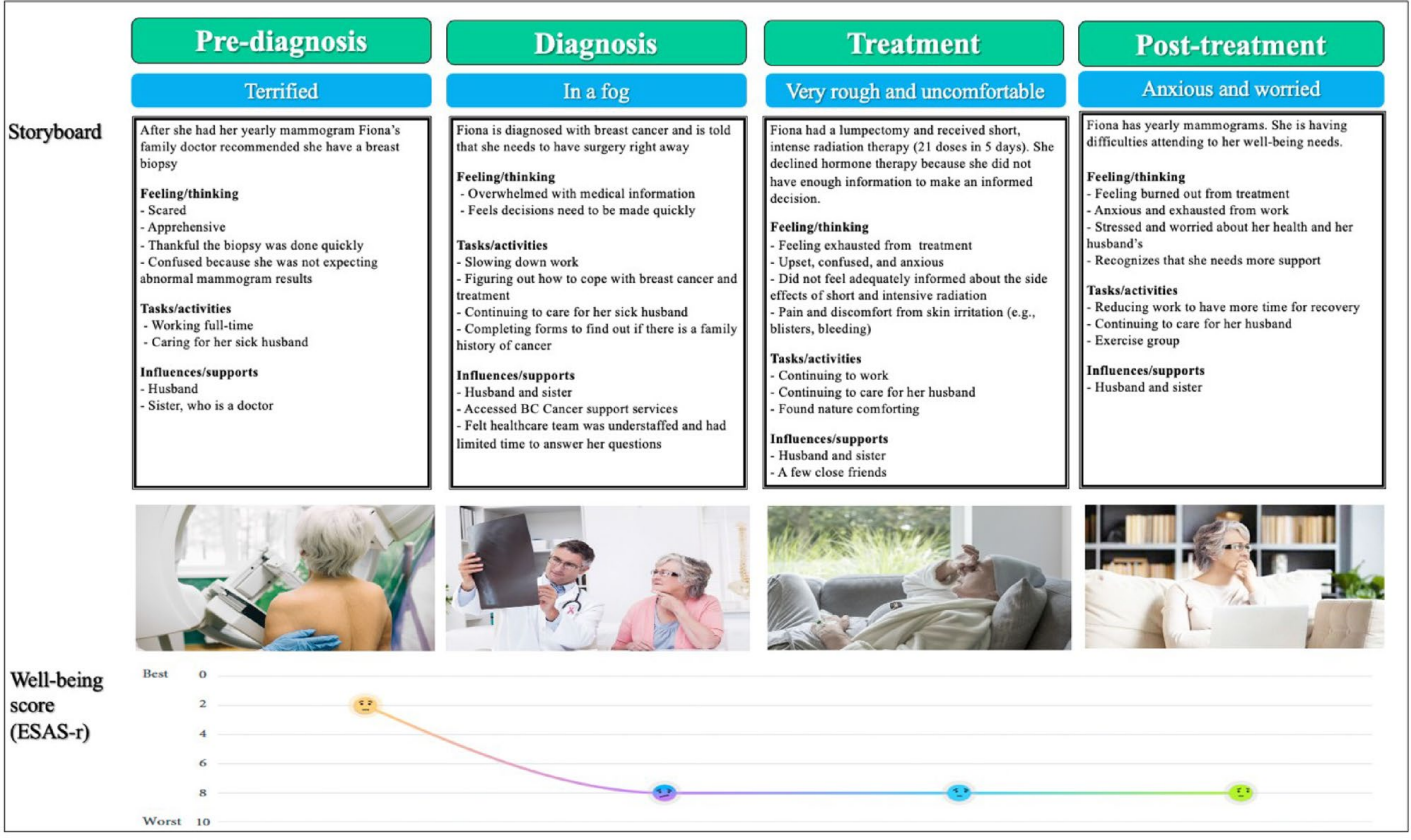
Example Journey Map of Fiona. Adapted from “Using journey maps to understand patient-reported outcome measures in the cancer journey”, by Kwon et al., 2024, *Canadian Oncology Nursing Journal*, 34 (4), 446. Copyright 2024 by Canadian Association of Nurses in Oncology

“Fiona” is a 69-year-old woman who received radiation therapy while simultaneously managing caregiving responsibilities for her spouse and experiencing financial strain due to employment challenges. Her well-being scores declined markedly during diagnosis and remained low through the post-treatment period, as visualized in her journey map (see Fig. 5). Fiona’s journey map illustrates an initial well-being rating of 2/10 in the pre-diagnosis phase which drops to 8/10 at diagnosis and remains low throughout treatment and post-treatment. The journey map contextualized her challenges related to her caregiving role and financial insecurity, illustrating that while her PROM score remains consistently low, the issues underlying it evolve, and may change over time. These insights inform a series of more tailored and meaningful prompts for clinicians during interactions with patients:

##### Pre-diagnosis

“You’ve rated your well-being fairly high. Can you share what is contributing to that, and what is impacting how you are feeling?”

##### Diagnosis

“Your well-being score has worsened significantly. Since your diagnosis, how have things changed for you? What’s been the most difficult to manage?

##### Treatment

“You continue to rate your well-being quite poorly. How has treatment been affecting you physically and emotionally? Are you getting the support you need at home? What does support look like to you?”

##### Post-treatment

“Your well-being scores suggest things haven’t improved since treatment ended. What do you think might help you feel better? Are there any supports you’d like to explore?”

These targeted questions, informed by Fiona’s journey map, allow clinicians to identify and address patients’ needs holistically—not just physical needs, but social and emotional as well. Fiona’s experience suggests the need for additional supportive care services such as financial counselling, mental health care, and caregiver assistance. By directly integrating PROM scores with individual narratives, journey maps serve as KT tools that can prompt clinicians to engage in meaningful dialogue with patients about the scores to subsequently anticipate emerging needs and address them through shared decision-making and consultations with other members of the healthcare team.

## Discussion and implications for practice

Personas and journey maps hold significant potential as educational KT tools for enhancing clinicians’ interpretation of PROMs. A systematic review by Wolff & team [2] identified a key barrier to the implementation of PROMs in that many clinicians lack the knowledge, skills, and confidence to interpret PROM scores and incorporate them into clinical decision-making. By integrating PROM scores into socially, psychologically, and emotionally grounded patient narratives, these tools transform numerical data into meaningful, contextualized narratives for interpreting care needs, particularly in complex clinical cases. These real-world examples of PROM scores and their interpretation help clinicians understand the unique meaning behind the scores and use them to tailor care plans that reflect individual patient needs and values. Our findings address a key limitation highlighted in Campbell et al.’s [4] systematic review, which noted that while PROMs can improve patient involvement and care quality, their impact is constrained when used without meaningful context or dialogue. Integrating PROM data into personas and journey maps reveals the nuanced, often invisible dimensions of patients’ lived experiences that numerical scores alone may obscure. The illustrated examples of “Trish” and “Fiona” demonstrate how personal values, life events, goals, and care transitions interact over time, influencing how individuals respond to care. These tools help clinicians to contextualize PROM scores, facilitating deeper reflection on what matters most to patients. For researchers and clinicians, personas and journey maps can serve as valuable resources for education and training to build clinicians’ self-efficacy in interpreting PROMs as entry points for therapeutic communication. Importantly, these tools are not intended for direct clinical use at the point of care. Instead, they are best suited for use in education and training contexts as tools to support clinicians in developing the knowledge, skills, and self-efficacy required to meaningfully integrate PROMs into practice. In simulation-based learning, PROM-integrated personas can facilitate critical reflection and structured debriefing, allowing learners to explore how diverse backgrounds shape health experiences, shared decision-making, and communication preferences, while also surfacing unconscious biases. Although the examples highlighted are drawn from oncology, these tools are broadly adaptable across clinical settings and patient populations.

### Lessons learned

Although personas and journey maps offer potential as KT tools for building clinicians’ abilities for integration of PROMs in clinical practice, their development requires intentional planning, facilitation, and sustained institutional support. Co-design for both personas and journey maps is inherently iterative and time-intensive, involving collaboration between patients, researchers, and facilitators to ensure relevance, rigour, and authenticity. In our project, we initially grouped participants by diagnosis when co-designing personas to aide workshop facilitation. This approach offered advantages by fostering shared understanding, building rapport, and enabling focused group work. However, it also posed limitations, particularly in capturing the breadth of individual experiences and the contextual nuances influencing PROM responses. To address this, we shifted to conducting individual interviews for journey map development, which allowed for more detailed exploration of diverse patient trajectories over time. While some may view the absence of a standardized co-design methodology as a limitation, we see this flexibility as a strength because our goal was not to generalize findings, but rather to highlight the complexity and uniqueness of individuals’ lived experiences to better inform the interpretation of PROM data.

Both co-design processes shared a common recruitment challenge of including participants from diverse social backgrounds, particularly those unfamiliar with digital platforms or disconnected from the healthcare system (e.g., individuals facing language barriers, limited access to technology, or reduced healthcare engagement). Given the virtual format of our worksops and interviews, we implemented strategies to foster inclusion, such as icebreaker activities at the beginning of sessions to help establish trust and openness, particularly when discussing sensitive or vulnerable topics. To minimize the identifiability of participants, they were encouraged to share perspectives that authentically reflected their own experiences, while also considering broader viewpoints or experiences that others in similar situations might face. These relational co-design processes rooted in dialogue and trust-building were essential to authentically capturing the diversity of patient experiences with PROMs.

Importantly, personas and journey maps must not be seen as definitive representations of patients. but as starting points for clinician reflection and meaningful patient-clinician therapeutic communication. Otherwise, clinicians may risk reinforcing stereotypes or reducing complex experiences to simplified narratives. The value of these tools lies not only in their content, but in how they are used – as education and training tools to build clinicians’ capacity and self-efficacy in integrating PROMs into practice. When thoughtfully implemented, these tools can illuminate broader social determinants of health (e.g., age, gender, income, and access to care) and their consequences on the interpretation of PROM scores. While PROMs are rigorously developed as reliable and valid standardized measures, their interpretation still requires contextual understanding. Reliability and validity ensure that PROMs consistently measure what they intend to across populations, but these standardized measures do not account for the social, emotional, or structural factors that shape how patients understand and respond to the questions. As a result, clinicians must interpret PROM scores within the context of each patient’s circumstances to meaningfully inform care. Personas and journey maps can support clinician education and training by illustrating how contextual factors shape PROM responses. However, these tools alone are insufficient to address micro, meso, and macro-level barriers to implementing PROMs in practice, such as limited time and competing clinical demands. To enhance PROM uptake in practice, additional implementation strategies are needed, such as workflow integration, leadership engagement, and audit-and-feedback to monitor and reinforce uptake [5]. While personas and journey maps cannot resolve all challenges associated with clinician adoption of PROMs [2], they offer valuable insights that help bridge the gap between standardized measurement and person-centred care. Their greatest strength lies not only in storytelling but in reshaping how clinicians understand, interpret, and act upon the lived realities behind PROM data to bring greater humanity and context to the numbers.

## Conclusion

Personas and journey maps are promising meaning-making tools to help clinicians interpret PROM scores to align with the person-centred approach to prioritize patients’ experiences, values, and goals. By integrating PROMs within narrative frameworks, these KT tools enable clinicians to learn to move beyond interpreting scores in isolation and instead engage with the broader social, emotional, and structural factors. This narrative integration fosters a more holistic understanding of patient needs and priorities. Importantly, such tools promote clinician reflection and support learning by making numeric data more tangible and actionable. As education/training KT tools, personas and journey maps integrated with PROMs can equip clinicians with the knowledge, skills, and self-efficacy to bridge the gap between standardized measurement and the realities of lived healthcare experiences.

## Data Availability

N/A.
